# PRISE2: Software for designing sequence-selective PCR primers and probes

**DOI:** 10.1186/1471-2105-15-317

**Published:** 2014-09-25

**Authors:** Yu-Ting Huang, Jiue-in Yang, Marek Chrobak, James Borneman

**Affiliations:** Department of Computer Science and Engineering, University of California, Riverside, CA USA; Department of Plant Pathology and Microbiology, University of California, Riverside, CA USA

**Keywords:** Selective PCR, Primer design, Probe design, 3’-end selectivity

## Abstract

**Background:**

PRISE2 is a new software tool for designing sequence-selective PCR primers and probes. To achieve high level of selectivity, PRISE2 allows the user to specify a collection of target sequences that the primers are supposed to amplify, as well as non-target sequences that should not be amplified. The program emphasizes primer selectivity on the 3’ end, which is crucial for selective amplification of conserved sequences such as rRNA genes. In PRISE2, users can specify desired properties of primers, including length, GC content, and others. They can interactively manipulate the list of candidate primers, to choose primer pairs that are best suited for their needs. A similar process is used to add probes to selected primer pairs. More advanced features include, for example, the capability to define a custom mismatch penalty function. PRISE2 is equipped with a graphical, user-friendly interface, and it runs on Windows, Macintosh or Linux machines.

**Results:**

PRISE2 has been tested on two very similar strains of the fungus *Dactylella oviparasitica*, and it was able to create highly selective primers and probes for each of them, demonstrating the ability to create useful sequence-selective assays.

**Conclusions:**

PRISE2 is a user-friendly, interactive software package that can be used to design high-quality selective primers for PCR experiments. In addition to choosing primers, users have an option to add a probe to any selected primer pair, enabling design of Taqman and other primer-probe based assays. PRISE2 can also be used to design probes for FISH and other hybridization-based assays.

## Background

Selective PCR is widely used in genetics, environmental sciences and medicine. A critical step in some applications, such as single-nucleotide polymorphism (SNP) analyses [1–4] and monitoring of environmental microorganism populations [5–7], is the isolation and amplification of specific DNA fragments, referred to as target sequences, from environmental samples. These samples are often complex mixtures of DNA fragments that may also include non-target sequences, which are pieces of DNA similar to target sequences but representing genes or organisms that are not the subject of the study. Naturally, such non-target sequences should not appear in the amplified product. A critical step in achieving such high-resolution amplification is the design of sequence-selective PCR primers, which are primers that amplify the target sequences but not the non-target sequences.

Designing such PCR primers manually is tedious and time consuming, and it can be streamlined and greatly simplified with the aid of appropriate software. Among other tasks, such software tools can be used to identify target and non-target sequences or to quickly evaluate the quality of a large number of primer candidates by computing their alignment to these sequences. They can also be used to compute biological parameters of the primers, like GC-content or melting temperature, that are essential for evaluating whether primers will produce specific amplification and high yield. These tools can also identify primers with appropriate complementarity properties, to prevent undesirable PCR side effects where primers bind to themselves by forming hairpin or primer-dimer structures.

The purpose of this document is to describe features of PRISE2 (PRImer SElector 2), a new software package for designing sequence-selective PCR primers and probes. PRISE2 is an extension of PRISE [8]. While preserving the main strategy of the design process and core algorithms from PRISE, PRISE2 contains a number of extensions and new features, including the ability to add probes to selected primer pairs, platform independence, and performance enhancements, among others.

In a nutshell, PRISE2 is an interactive software package for designing primers and probes for PCR experiments, that accepts sets of target and non-target sequences on input, and produces primer pairs that will typically selectively amplify the target sequences. Customizable features and settings are available to ensure that the computed primers will be effective for the application at hand.

One distinctive feature of PRISE2 is that it allows users to emphasize primer selectivity at the 3’end. This feature is essential for selective amplification of conserved sequences such as rRNA genes, and to accurately differentiate between target and non-target sequences during the actual laboratory PCR process [9–11]. The need for such precise selectivity arises when amplifying sequences from mixtures of DNA. Examples of such applications include subtype analyses, in which very similar groups of genes need to be distinguished, or rRNA gene studies to monitor population levels of specific microorganisms in environmental samples that contain millions of different organisms [5–7]. In fact, PRISE2 (as well as its predecessor PRISE) was designed specifically to support our studies of microbial communities [12–17], that often involve analysing environmental samples containing mixtures of both target and non-target sequences. Another application where such high selectivity is useful is genomic walking [11, 18–20]. The focus on the 3’ end selectivity is implemented through a detailed and customizable per-position mismatch allowance matrix that sets more stringent mismatch criteria at the 3’ end than for the rest of the primer. We are not aware of any other software that provides such sophisticated selectivity settings.

When searching for candidate primers, PRISE2 uses a custom algorithm that assigns appropriate mismatch weights, mentioned earlier, to different positions when aligning candidate primers against their potential binding regions. Other programs typically use local alignment tools, such as BLAST, to do similarity searches to identify the binding position of primers, which may result in the loss of some match information over the entire primer range, especially when the match is not perfect toward the primer ends.

Another distinctive feature of PRISE2 is the probe design function, which is useful in quantitative analyses to measure the amplification of target sequences. For example, it enables the design of Taqman and other primer-probe based assays, or probes for FISH and other hybridization-based assays. The probe design feature is integrated with the primer design process, in the sense that users can add probes to selected primer pairs, and the triples consisting of a forward primer, reverse primer and a probe are evaluated in tandem, in terms of coverage and other quality criteria. When designing probes, users can specify biological parameters that may be different than those for primers. For example, setting the melting temperature ranges for probes and primers at different levels helps to ensure proper functioning of probes during the Taqman PCR process. The mechanism for defining mismatch criteria for probes is different than that for primers, with mismatches near the middle of the probe given more weight than at the ends. For this reason, the algorithm for selecting probes is also quite different than that for primers.

Unlike some other programs [21–25], PRISE2 does not require signature primers. (Signature primers are short pieces of DNA that are only conserved in target sequences.) Using signature primers reduces computational cost; however, not all groups have unique signatures [25]. PRISE2 considers all combinations of forward/reverse primers and probes, so for such groups it is able to find individually non-specific but group-specific primer sets, for which the methods using signature primers are not likely to be effective.

PRISE2 also differs from existing tools in terms of the fundamental approach to the design process, by emphasizing interaction with the user, in order to take advantage of human expertise. Rather than simply computing a primer-probe set that optimizes user-specified criteria, at several stages of the process PRISE2 produces a list of candidate primers or probes, each with a list of quality indicators, like coverage, length, GC-content, and other. The user can manipulate these lists, sorting them according to different criteria, to choose a subset of candidates to proceed to the next stage. This interactive approach also allows users to backtrack through the process to tune the program’s parameters. For example, if insufficiently many primer candidates are produced, due to excessively stringent biological settings, users can go back to the previous step and experiment with different parameters to increase the number of candidate primers.

More extensive comparison between PRISE2 and other primer design tools can be found in Results and Discussion section later in the paper.

## Implementation

### Overview of the design process

As explained in the background section, the primer design process in PRISE2 is essentially the same as in its predecessor, PRISE. The module for probe design is new. We describe the complete process in this section.

Designing primers and probes in PRISE2 is accomplished in three stages:

1. *Identification of target and non-target sequences*. Here, the user can download a collection of sequences from GenBank and use the provided interactive tools to choose from them (or from other collections of sequences) the desired sets of target and non-target sequences.
2. *Generation of candidate primer pairs*. In this step, the program computes a set of primer candidates, according to the specified parameters, and groups them into primer pairs. Then the user can use a variety of sorting tools to manipulate the list of these primer pairs to choose a smaller collection of high quality primer pairs.
3. *Adding probes*. Once these desired primer pairs have been selected, the next module of PRISE2 allows probes to be added to each primer pair. For each primer pair, PRISE2 determines a list of candidate probes. This produces a collection of primer-probe sets that can be sorted according to multiple criteria, thus allowing the user to choose, ultimately, the final collection of primer-probe sets best suited for their PCR experiments.

Below we give a detailed description of these steps.

### Creating lists of target and non-target sequences

In this step, users first need to identify the seed sequences and create the hit table. The seed sequences are DNA sequences that the user aims to amplify, and the hit table is a file, created by subjecting the seed sequences to an analysis using BLAST (blastn) [26], that contains summary information (species/organisms/GI, etc.) for similar or related sequences from all species or organisms in specified databases. Users have two options to run BLAST, either remotely through the NCBI website, following the instructions provided in PRISE2, or locally, if they have an installation of BLAST on their machine. In the latter case, PRISE2 provides an interface to integrate sequence identification with a locally installed BLAST application and databases.

Once the seed sequences and the hit table are identified and saved, PRISE2 will download associated records from GenBank and perform pairwise similarity (percentage identity) analyses between these sequences. Those sequences are then displayed as a list, and can be interactively manipulated and sorted according to specified properties, similarity to seed sequences, or various other attributes. This allows the users to identify the desired target and non-target sequences, which then can be saved for future use. In a typical application, the sequences most similar to seed sequences would be designated as target sequences, while non-target sequences would be selected from the remaining set. Other sequence attributes, provided by PRISE2, may be useful in this task, for example sequence length.

### Designing primers

To design primers, the user needs to specify first the sets of target and non-target sequences. In most situations, these sequences are those identified in the previous step, but this is not a requirement. Some users may wish to use their own collections of target or non-target sequences, whether obtained earlier from PRISE2 or from some other source. PRISE2 will accept any collections of sequences as long as they are in the FASTA format.

Next, the user can specify desired properties of individual primers and of pairs of primers. These properties include, for example, primer length, GC content or melting temperature. Among other options, users can also restrict complementarity properties of primers, to assure that chosen primer candidates do not bind to themselves and that, in selected primer pairs, the forward primer does not bind to the reverse primer. For convenience, all parameters have pre-tested default choices that are likely to work well in most typical applications.

The following step involves choosing the primer selectivity settings. The purpose of selectivity settings is to identify highly selective primers, namely those that will bind to most target sequences but to as few as possible non-target sequences. These settings allow users to define what constitutes a match between a primer and a sequence, and they can be controlled separately for target and for non-target sequences. Roughly, stringent settings correspond to nearly perfect matches, while more flexible settings represent inexact matches. Thus more stringent settings produce fewer primer candidates than relaxed settings. As in the previous steps, typical users will likely use the default settings only. However, PRISE2 also allows the users to customize these parameters, either through a basic interface where they choose stringency levels, or more advanced options where they can manually adjust the values of all parameters.

There are two categories of selectivity settings: *mismatch allowance mechanism* and *mismatch cost matrix*. The purpose of mismatch-allowance mechanism is to emphasize primer selectivity on the 3’ end, which is crucial for functional primers, as explained earlier. This is accomplished by setting limits on the accumulated number of mismatches, starting at the 3’ end and ending at any position. For example, one can specify 0 mismatches on the first 3 positions, at most 1 mismatch on the first 5 positions, and at most 2 mismatches on the first 7 positions.

The mismatch cost matrix is a nucleotide-to-nucleotide dissimilarity function that reflects the likelihood of binding to occur, with smaller values representing higher likelihood. For example, in its simplest form, its entries could be 0 for equal bases (matches) and 1 for different bases (mismatches). Users can adjust these values to distinguish different types of mismatches, for example A/G and A/C mismatches may be assigned different costs.

Consider a primer candidate *p* and a template string *t* (that is, *t* is either a target or a non-target sequence). For a fixed location *l* in *t*, let *t’* denote the substring of *t*, of the same length as *p*, starting at location *l*. For all locations in *p*, PRISE2 computes accumulated costs of mismatches (using the mismatch cost matrix) starting from the 3’ end of *p*, and if any of these values exceeds the corresponding mismatch allowance, it determines that there is no match at location *l*. Otherwise, it considers position *l* as a potential match and it stores the total accumulated mismatch cost as the cost of aligning *p* and *t’* at location *l*.

By computing such alignment costs for all potential match locations *l*, PRISE2 then chooses among them the most likely location in *t* for this primer *p* to bind, simply by choosing the location with minimum cost. (This computation is accomplished in PRISE2 with a custom string similarity algorithm specially designed for this purpose.) In cases when no potential match locations in *t* are found, PRISE2 determines that *p* does not bind to *t*.

PRISE2 has also an option to allow gaps in alignments. This is implemented by assigning costs to gaps and putting a limit on the number of gaps. In the computation described above these costs are included as costs of nucleotide insertions or deletions.

Repeating the above process for all primer candidates *p* and target/non-target sequences *t*, PRISE2 will determine which primers bind to which sequences and at which positions.

At this point, PRISE2 will match all primer candidates into pairs. These pairs are then presented to the user, sorted according to their selectivity measure, that is computed as follows. Consider a pair *f*, *r* of primers. Let *cov*_*t*_*(f,r)* denote the coverage of this primer pair in target sequences, and let *cov*_*n*_*(f)* and *cov*_*n*_*(r)* denote the coverage of *f* and *r*, respectively, in non-target sequences. These values are normalized so that they are in the range [0,1]. The selectivity of this primer pair is then computed as


Note that this will produce high values for pairs *f*, *r* that jointly match many target sequences, but where each of *f* and *r*, individually, matches few non-target sequences. To emphasize, this value is used only to sort the primer pairs in the display window, to give the user a rough estimate of the quality of primer pairs.

The number of candidate primer pairs depends on the stringency of all settings. If too few or too many pairs are produced, PRISE2 allows the user to backtrack and adjust these settings to re-compute new candidate pairs.

After the user is satisfied with the list of candidate pairs, he/she can review their quality criteria and, based on these, he/she can select a small number of primer pairs to be used in the experiment, or to proceed to the probe design stage. To assist users with this task, the list of primer pairs is displayed in a tabular format that shows all quality indicators for these primer pairs, including the percentages of target and non-target sequences that the primers are predicted to bind to, the product length, GC-content, complementarity properties, etc. For each primer pair, users can even display and examine the alignments between this pair and all target or non-target sequences. An easy to use graphical interface allows the users to manipulate this list, in an interactive fashion, including removing primer pairs, adding (manually) new primer pairs, and sorting the list according to a wide variety of criteria.

### Designing probes

Once the user identifies and selects a collection of primer pairs of interest, PRISE2 provides an option of adding probes to these pairs. This module can be also used for designing probes for FISH and other hybridization-based assays. In quantitative PCR (qPCR) analyses such as Taqman assays, probes play an essential role in monitoring the amount of sequence amplification.

The process of probe design is similar to that for primers. It involves selecting probe properties, such as the probe length, GC content, etc. One probe attribute that assures that the probes will function properly during the qPCR process is the difference of melting temperatures between the primer pair and its associated probe. For example, for Taqman assays, setting the melting temperature for probes higher than for the primers ensures that the probe will bind to the target before the primers. This sequential binding is essential for consistent quantification in such assays, because measurement of amplification events occurs when DNA synthesis from the primer leads to the polymerase enzyme contacting and then cleaving the quencher located on the end of the probe, resulting in a detectable fluorescent signal.

As in the primer design process, users can specify their own scoring scheme for probes, the cost function, and the mismatch allowance matrix, to obtain the desired selectivity to target sequences. Unlike for primers, however, where matches on the 3’ end were considered more important, in the case of probes, PRISE2 focuses on the middle section of the probe by allowing the user to specify the number of continuous matches near the centre of the probe. Such continuous matches increase the likelihood of binding, even if some mismatches occur near the ends of the probe. These mismatch criteria can be specified separately for target and non-target sequences.

Once probe properties and selectivity settings are defined, PRISE2 will compute, for each pair of selected primers, the list of probes that match all the criteria.

The final result is a list of primer-probe sets, each consisting of a pair of primers and a probe, that meet all criteria for primers and probes. PRISE2 displays these results in a window with separate tabs for each primer pair, sorted according to the selectivity function:


In this formula, *f*, *r*, and *p* denote the forward primer, the reverse primer, and the probe in a probe set, and *cov*_*t*_*()* and *cov*_*n*_*()* represent the normalized coverage values in target and non-target sequences, respectively. For each primer pair the program shows a tabulated list, with rows corresponding to probes and columns showing properties of the corresponding primer-probe set. As for primers, these lists can be manipulated and sorted by the user, according to a number of different criteria. Ultimately, using these tools, users can determine a small number of primer-probe sets to be used in their PCR experiment.

More detailed description of the primer/probe design process can be found in the PRISE2 manual and tutorial, both provided on the PRISE2’s web page.

## Results and discussion

### Experiment

To demonstrate that PRISE2 finds highly selective, functional primer-probe sets, we used PRISE2 to design two Taqman real-time qPCR assays, which were subsequently tested in a laboratory experiment. Primer and probes sets were designed to differentiate the rRNA ITS region of two closely related strains of the fungus *Dactylella oviparasitica* (DO50 and DO60). Sequence-selective primers and probes were designed using PRISE2 with the default settings. The non-target sequence used for the design of the DO50 assay was DO60, while the non-target sequence used for the DO60 assay was DO50.

**Figure 1:**
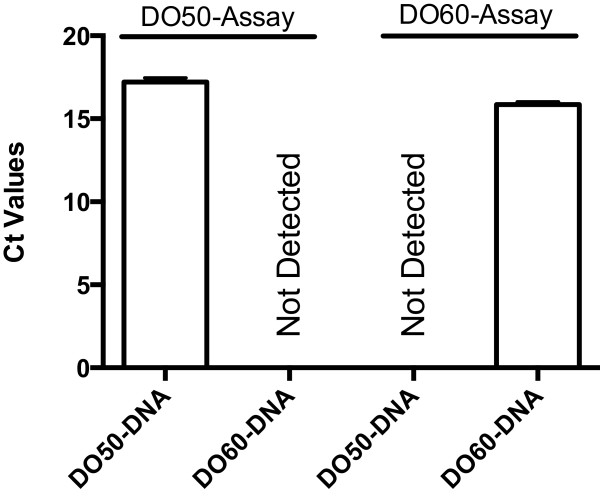
**Experimental results for DO50 and DO60 assays.** qPCR of DO50 and DO60 DNA using sequence selective assays for DO50 and DO60. qPCR was performed using a Bio-Rad iCycler MyiQTM Real-Time Detection System (Bio-Rad Laboratories, Hercules, CA, USA). The selective primers for the DO50 and DO60 assays are DO-50 F1 (ATCGGCCTCACAAA) and DO-50R1 (TAACCAATTCCTTGTTGTT) and DO-60 F2 (AGCGAAACCCTCTCA) and DO-60R2 (TACGAGTTGTCGCAATAC), respectively. The selective probes for the DO50 and DO60 assays are DO-50Probe-1, [6-FAM]AACAGCACAGTGGACCTGCC[BHQ1a-6FAM] and DO-60Probe-2, [6-FAM]AAAGCTAGCGGGCACAGGC[BHQ1a-6FAM], respectively, where BHQ1a is Black Hole Quencher 1 (Eurofins MWG Operon, Huntsville, AL, USA). The targets are fragments of the ITS rRNA gene with sizes of 94-bp and 75-bp for DO50 and DO60, respectively. The thermal cycling conditions were 94°C for 5 minutes; 42 cycles of 94°C for 20 seconds, X°C for 30 seconds and 72°C for 30 seconds; followed by 72°C for 10 minutes; where X = 58.3 for DO50 and 63 for DO60. Amplification reactions were performed in iCycler iQ PCR Plates with Optical Flat 8-Cap Strips (Bio-Rad Laboratories). PCRs were performed in 25-μl reactions containing the following reagents: 50 mM Tris (pH 8.3), 500 μg/ml bovine serum albumin (BSA), 2.5 mM MgCl_2_, 250 mM of each dNTP, 400 nM of each primer, 250 nM of the probe, 8.36 x 10^6^ copies of the ITS rRNA gene, and 1.25 units of Taq DNA polymerase. Ct = threshold cycle. Error bars indicate standard error. n = 4 for each column.

The assay for DO50 produced a robust amplification signal from DO50 DNA, but no signal from the DO60 DNA. A similar but opposite result was obtained for the DO60 assay (see Figure 1 for details). These results demonstrate the ability of PRISE2 to create useful primers and probes for sequence-selective assays.

Primers designed with PRISE/PRISE2 have been already used in multiple investigations of microbial community composition conducted by the last author (JB) and his group [12–17]. These studies typically require amplifying targeted sequences from DNA mixtures extracted from environmental samples containing hundreds to thousands of non-target sequences, and the high resolution of the primers produced by PRISE/PRISE2 tools and their ability to distinguish between target and non-target sequences played critical role in these studies. For example, in a study examining the role of microorganisms in inflammatory bowel disease (23), use of PRISE enabled measurements of specific bacterial rRNA gene sequences in a habitat harbouring hundreds or thousands of different microorganisms, providing putative links between specific host-microbe interactions and disease causation.

### Comparison with PRISE

In comparison to its predecessor PRISE, the most significant new feature of PRISE2 is the probe design option that can be useful for Taqman and other primer-probe assays. In quantitative analyses such as Taqman assays, probes can be used to measure the amplification of target sequences along with a pair of primers. Probes can also be designed for FISH and other hybridization-based assays.

Unlike PRISE, which is only available on Windows, PRISE2 is a cross-platform software, not restricted to a specific operating system. It is written in C++, with the GUI implemented with the Qt toolkit. We provide PRISE2 installations for Windows, Macintosh and Linux machines.

Another new feature in PRISE2 is the option to communicate with a local BLAST application, which provides a more convenient way to identify target and non-target sequences, without the need for connecting to the NCBI website.

Although the core algorithms for sequence similarity and identifying primer candidates remain the same, several performance improvements were made to speed up the computation for large data sets. For instance, the module for computing primer candidates is roughly 4 times faster in PRISE2 than in PRISE. When run on a data set of 123 target and 319 non-target sequences from NCBI flu database, each of length approximately 1000 bps, with most parameters at default values, PRISE2 completed this task in 12 minutes, compared to 48 minutes in PRISE (on a Windows 8 machine with 8 GB memory and 2.4 GHz CPU).

### Comparison with other software

PRISE2 implements a flexible mechanism for specifying primer-template mismatch criteria at specific positions at the 3’ end, with a detailed per-position mismatch allowance matrix. One other tool that allows users to differentiate selectivity properties of the 3’ and 5’ ends is PrimerProspector [27], where different weights can be applied to the 3’ and 5’ ends of primers to estimate the likelihood of binding. To consider a primer candidate as functional to a specific template, PrimerProspector requires this candidate to have a number (user-specified) of continuous base matches starting at the 3’ end. This feature can be thought of as a restricted case of PRISE2’s 3’-end selectivity mechanism. PRISE2 gives the user more control than PrimerProspector, to cover scenarios where primer-template binding is likely to occur even in the absence of continuous matches at the 3’ end. In addition, unlike PRISE2, PrimerProspector does not use non-target sequences.

PRISE2 allows users to interactively design probes for selected primer pairs. Primer3plus [28] is a widely used web-based program for primer design, which provides similar options. In Primer3 users can design a primer set consisting of a forward primer, a reverse primer and a probe, and they can choose a number of settings for controlling the quality of the primers, similar to PRISE2. Primer3, however, focuses on designing primer sets targeting single sequences, and it does not take non-target sequences into consideration, nor does it allow mismatches to be placed on the 3' ends of the primers. In Primer3, the design is a single-shot process, without the flexibility and convenience of the interactive procedure available in PRISE2.

Primer-BLAST [29] is an integrated tool for primer design provided by NCBI. It combines Primer3 and BLAST along with the Needleman-Wunsch (NW) global alignment algorithm, which overcomes the shortages of local alignment for primer design purpose. Primer-BLAST first utilizes Primer3 to generate the candidate primer pairs, and then applies the specific BLAST to check specificity. Since it consists of two separate modules, users can check the specificity of existing primer candidates. However, Primer-BLAST inherits limitations of Primer3. It does not have the feature to design a desired set of non-target sequences, and it has only limited ability to control mismatch positions. Primer-BLAST achieves specificity of primers by forcing the number of mismatches between primers and unintended targets from a user-specified database, in a specific region at 3’ end.

Other existing software packages for primer design include QuantPrime [30] and PRIMEGENS [31, 32]. Both these tools do not include options to specify non-target sequences or to design probes. For the 3’ end selectivity, QuantPrime provides only a rudimentary mechanism of limiting the number of mismatches near the 3’ end, while PRIMEGENS does not implement this feature.

## Conclusions

In this paper we presented PRISE2, a new interactive software package for designing sequence-selective PCR primers. PRISE2 facilitates the design process through an intuitive, user-friendly graphical interface. Among its main features, PRISE2 emphasizes primer selectivity at the 3’ end and it has the capability to add probes to selected pairs of primers. Pre-tested default choices for all parameters are provided to simplify the process for less experienced users; while those more experienced can use the advanced options where these parameters can be highly customized. The package also includes a manual and a tutorial, to assist users with the design process.

PRISE2 has been tested on two very similar strains of the fungus *Dactylella oviparasitica*, DO50 and DO60, and it was able to create highly selective primers and probes for each of them. This result demonstrates the ability of PRISE2 to create high-quality sequence-selective assays.

## Availability and requirements

PRISE2 can be downloaded from http://alglab1.cs.ucr.edu/OFRG/PRISE2.php. Alternatively, one can go to the OFRG website at http://algorithms.cs.ucr.edu/OFRG and follow the link to PRISE2. The program is distributed as an executable file that can be executed once downloaded and no additional installation is required.

PRISE2 is free for non-commercial use. Information about the commercial use of PRISE2 can be found at that website.

PRISE2 requires a minimum of 512 MB of RAM (1 GB of RAM or more is recommended). The software can run on the following systems:

-  Windows 2000/NT/XP/7/8
-  Ubuntu 9.04 or higher
-  Mac OS 10.5 or higher

**Note:** To run PRISE2 on Mac OS X Mountain Lion or higher, users may be required to change or bypass their Gatekeeper settings to allow the installation. Detailed information about this process can be found at http://www.imore.com/how-open-apps-unidentified-developer-os-x-mountain-lion.

Activated Internet connectivity is needed for full functionality, to connect to the NCBI website. For users who have target and non-target sequences already selected, or who have a local installation of BLAST, no Internet connection is required.

PRISE2 is an open-source project. Users interested in obtaining the source code should contact the authors by email.

PRISE2 is written in C++, with the graphical user interface implemented with the Qt toolkit.
